# The Indeterminate Form of Chagas Disease

**DOI:** 10.5935/abc.20180027

**Published:** 2018-02

**Authors:** Victor Sarli Issa

**Affiliations:** Instituto do Coração (InCor) do Hospital das Clínicas da Faculdade de Medicina da Universidade de São Paulo; Hospital do Coração (HCor), São Paulo, SP - Brazil

**Keywords:** Chagas Disease/physiopathology, Chagas Cardiomyopathy, Ventricular Dysfunction, Endomyocardial Fibrosis, Diagnostic Imaging, Epidemiology

The presence of diffuse fibrosis in the myocardial tissue is a characteristic of Chagas heart disease.^1^ The mechanisms proposed to explain such fibrosis areas vary and include direct injury by *Trypanosoma cruzi* to the cardiac tissue, as well as tissue ischemia due to microcirculation changes and microvascular thrombosis mediated by inflammatory^2^ and immune^3^ processes. The myocardial fibrosis not only reveals important aspects of the pathophysiology of the disease, but has a clinical significance,^4^ because its progression can lead to injury to the heart conduction system, contributing to generate arrhythmia, as well as systolic and diastolic ventricular dysfunction, in addition to favoring the appearance of thromboembolic phenomena from the hypokinetic or akinetic areas.

This issue of the *Arquivos Brasileiros de Cardiologia* presents the results of a study jointly conducted by three different centers in the city of Salvador, Bahia state, about the clinical significance of the fibrosis found in patients with Chagas disease, in both the indeterminate and heart disease (with and without left ventricular dysfunction) stages. The search for fibrosis was performed by use of late enhancement cardiac magnetic resonance imaging. The authors have reported late enhancement compatible with fibrosis in 41% of the patients with the indeterminate form, a figure similar to that found in patients with heart disease without ventricular dysfunction. In addition, it is worth noting the similar findings in the other groups regarding the clinical characteristics and the levels of type B natriuretic peptide, troponin, interleukins 2, 4, 6 and 10, tumor necrosis factor alpha and gamma interferon.^5^

Previous studies have identified myocardial fibrosis in patients with Chagas disease and correlated its intensity with the severity of ventricular dysfunction and symptoms. A study of 51 patients with Chagas disease using late enhancement technique has identified images compatible with myocardial fibrosis in 20% of the 15 patients with the indeterminate form.^6^ Similar results have been found by using other imaging techniques: a study of 40 patients with the indeterminate form of Chagas disease, using echocardiography and single photon emission computed tomography (*gated-*SPECT) myocardial perfusion imaging, has detected some changes in perfusion and myocardial motion in 25% of the individuals, including perfusion defects, reduced ejection fraction and intraventricular dyssynchrony.^7^

The finding by Rabelo et al.^5^ of similar phenotypes in patients with the indeterminate form and those with heart disease (and normal left ventricular function) draws attention to the discussion on the meaning of the indeterminate form definition. This concept has been applied to patients with positive serology for *Trypanosoma cruzi* and neither gastrointestinal disease nor myocardial injury identified on clinical assessment, chest X-rays and electrocardiogram. However, the value of that definition has been questioned based on the current methods to assess cardiac function and morphology. One way to estimate the value of those findings is to assess the long-term outcome of patients.^8^ A study from 2001 of 160 patients with the indeterminate form, followed up for 98 months, and based on clinical, electrocardiographic and echocardiographic findings (two-dimensional and M mode) has reported stable ejection fraction during follow-up despite the appearance of electrocardiographic changes.^9^ A study with a 10-year follow-up of blood donors with positive serology for *Trypanosoma cruzi* has estimated the incidence of the progression to heart disease in 1.85 per 100 individuals-year, with heart disease diagnosis based on electrocardiographic and two-dimensional echocardiographic changes.^10^ However, studies assessing the long-term follow-up of patients with the indeterminate form of Chagas disease by using the currently available techniques for analysis of myocardial function and morphology and mortality data still lack.

Finally, despite the progression over the last decades of the methods to identify the patients at higher risk or with subclinical morphological changes, the likelihood of the patients' prognostic improvement still faces the limitations of therapy, especially considering the negative results of the etiological treatment of Chagas disease's chronic forms.^11^ Those and other difficulties that persist in the management of patients with Chagas disease are a constant challenge for the doctors and researchers who cope with such a severe condition.
